# Fatigue in early, intensively treated and tight-controlled rheumatoid arthritis patients is frequent and persistent: a prospective study

**DOI:** 10.1007/s00296-018-4102-5

**Published:** 2018-07-16

**Authors:** Margot J. M. Walter, T. M. Kuijper, J. M. W. Hazes, A. E. Weel, J. J. Luime

**Affiliations:** 1 Department of Rheumatology, Erasmus Medical Center, University Medical Center Rotterdam, Postal box 2040, 3000 CA Rotterdam, The Netherlands; 2 Department of Rheumatology, Maasstad Hospital, Rotterdam, The Netherlands

**Keywords:** Early RA, Fatigue, Patient-reported outcomes

## Abstract

Fatigue has a large impact on quality of life and is still unmanageable for many patients. Study aims were describe (1) the prevalence and pattern of fatigue over time in patients with early rheumatoid arthritis under a treat-to-target strategy and (2) identify predictive factors for worsening and recovering of fatigue over time. Data from the tREACH study were used, comparing different treatment strategies with fatigue as secondary objective. Patient outcomes on fatigue, quality of life, depression, and coping were obtained every 6 months and clinically assessed every 3 months. Prediction of fatigue at 12 months was investigated with an ROC curve. Analysis was stratified into non-fatigue and fatigue at baseline. Logistic regression was used for the evolution of fatigue in relation with the covariates over time. Almost half of all patients (*n* = 246) had high fatigue levels at baseline, decreasing slightly over time. At 12 months, 43% of patients were fatigued; while 23% of the initially fatigued patients showed lower levels of fatigue, the fatigue level had increased in 15% of the initially non-fatigued patients. The strongest predictor of fatigue was the previous fatigue levels (AUC 0.89). Higher score on the depression scale and coping with limitations was associated with developing fatigue over time in the initially non-fatigued group. Despite a strict treat-to-target strategy, fatigue remained an overall problem during the first year of treatment, and was mainly predicted by its baseline status. In subgroups, a small additional effect of depression was seen. Monitoring fatigue and depression may be important in managing fatigue.

## Background

Studies have shown good results concerning remission and structural radiographic damage of the joints by tight control and treat-to-target management [1, 2]. Despite these effective strategies and reaching remission of disease activity, patients with rheumatoid arthritis (RA) still may experience a burden of the diseases like pain and fatigue [3]. From 40 to 80% of the RA patients are fatigued, which may affect their lives [4–6]. The impact of fatigue permeates through every aspect of their lives, limiting work participation [7], family activities or social activities [8], sports and simply enjoying life as it is.

Thus, fatigue is an important aspect for many patients with a high impact on patients by influencing the choices they make in their social life. Moreover, fatigue is associated with a reduced health-related quality of life and depression [8, 9] and is the most limiting factor for the ability to work [10]. Because of this large impact, it is important to study fatigue. So far, little is known about fatigue during the disease course in early RA. According to patients, reducing fatigue is an important treatment target, but is not often addressed during consultations [11, 12].

The evolvement of fatigue over time in patients with early RA had been addressed in a few previous studies. An 8-year study from The Netherlands suggested little change of fatigue levels over time at group level, while individual levels fluctuated over time [13]. In contrast, a study in early RA revealed an improvement in fatigue for 40% of the patients, while fatigue levels increased in another 24% [14]. These were both cohort studies in which treatment was left to the discretion of the physicians.

Therefore, the objective of the present study was (1) to describe the prevalence and pattern of the fatigue over time in patients with early RA under a treat-to-target strategy and (2) to identify predictive factors for worsening and recovering of fatigue over time.

## Methods

### Study participants

Data from the tREACH study (treatment in the Rotterdam Early Arthritis Cohort, 2007–2013), comparing different treatment strategies with fatigue as secondary objective, and patients fulfilling the ACR-EULAR 2010 criteria for RA, were used for this analysis [15]. This multi-centered trial compared different initial treatment strategies in early RA patients. Inclusion criteria for the tREACH study were: age ≥ 18 years, arthritis in one or more joint(s) and symptom duration < 1 year. Patients were recruited from the outpatient clinics of all participating centres between July 2007 and April 2011. Initial treatment arms were: I methotrexate, sulfasalazine, and hydroxychloroquine (HCQ) + glucocorticosteroids (GCs) intramuscularly; II methotrexate, sulfasalazine (SASP), and HCQ + oral GC-tapering scheme; and III MTX + oral GC. Treatment was escalated to biologicals if DAS44 > 2.4 [16–18]. Details can be found in Claessen et al. [19]. The medical ethics committee at each participating center approved the study protocol and all patients gave written informed consent before inclusion (METC 2006-252, trial protocol number 2006-005771-18).

### Data collection

Patients’ demographic and clinical characteristics as well as the frequency of erosions were recorded at baseline. Disease activity measures and adjustments to treatments were applied every 3 months. Fatigue, coping strategies for pain and physical limitations, health-related quality of life, and symptoms of anxiety and depression were assessed every 6 months.

### Clinical evaluation of disease activity

The disease activity was assessed by the disease activity score (DAS28), which is a composite score assessing swollen joints, tender joints, and the erythrocyte sedimentation rate (ESR), and includes a Visual Analog Scale (VAS) global (range 0–10). Higher score indicates a higher disease activity [20]. To investigate the relation with fatigue and painful joints, we used the separated tender joint count (TJC 44).

### Patient-reported outcome measures

#### Fatigue

Fatigue level was measured by VAS and the Fatigue Assessment Scale (FAS). The VAS (100 mm) fatigue involves the severity of the fatigue over the past week with the anchors: no fatigue (0 mm) and extremely fatigued (100 mm). The scale is sensitive to change, valid, and reliable, but no cut-off point has been determined [21, 22]. The FAS is a ten-item fatigue scale with a good internal consistency, reliability, and validity [23, 24]. Five questions reflect physical fatigue and five questions reflect mental fatigue. The instruction is directed at how a person usually feels. Each item is scored on a five-point rating scale ranging from 1 ‘never’ to 5 ‘always’. The total scores thus range from 10 to 50 and are interpreted as follows: 10–21 no fatigue; ≥ 22–34 substantial fatigue; and ≥ 35–50 extreme fatigue [25, 26].

#### Disease-related

The Rheumatoid Arthritis Disease Activity Index (RADAI) measures self-reported disease activity [27]. It contains five items: global disease activity during the last month, today’s disease activity in terms of swollen and tender joints, and today’s severity of arthritis pain and stiffness and self-assessed tender joints. It is measured on a scale ranging from 0 to 10, where higher scores indicate more disease activity [28].

#### General health

The health-related quality of life (HRQOL) was scored with the SF-36 (range 0–100). A higher score indicates a better HRQOL. It assesses eight health concepts: physical functioning, bodily pain, role limitations due to physical health problems, role limitations due to personal or emotional problems, emotional well-being, social functioning, energy/fatigue, and general health perceptions which are summarized in a physical component summary (PCS) and mental component summary (MCS) score [29].

#### Psychosocial

Coping was measured via the Coping with Rheumatoid Stressors (CORS) scale. The subscales dealing with pain, decreasing activities (range 8–32), and limitations (range 10–40) were included in tREACH study. A higher sum score indicates more frequent use of the coping strategy. Both subscales have good internal consistency and high test–retest reliability [30, 31].

Depression and anxiety were measured by the Hospital Anxiety and Depression Scale (HADS). Two subscales with each seven items are calculated with higher scores indicating more severe symptoms of anxiety or depression [32]. Categorical scores are available. Scores between 0 and 7 represent ‘no case’; 8–10 ‘possible case’; and 11–21 ‘probable case of anxiety or depression’ [32, 33].

### Statistical analysis

Simple descriptive techniques were used to describe the prevalence of fatigue and its associations with other covariates at baseline. Mean and SD or percentages were described, as appropriate. As longitudinal fatigue evolvement was diverse, we stratified the analysis into two clinically relevant patient samples: those with no fatigue (FAS values 10–21) and those with fatigue (FAS values 22–50) at baseline [25, 26]. The baseline differences between fatigued and non-fatigued patients among continuous variables were tested with the unpaired *t* test or Mann–Whitney *U* test as appropriate. Categorical variables were tested using Pearson’s Chi-square test.

Prediction of fatigue at 12 months was investigated with an ROC curve with fatigue as a continuous variable. To investigate variables that are important for change of fatigue over time, logistic regression analyses predicting fatigue status at 12 months by baseline covariates were performed for each stratum. First, univariable analyses were performed. Thereafter, starting with full models, backward elimination was performed until all remaining variables reached a significance level of *p* < 0.10. Age and gender were forced into the models regardless of their levels of significance. Missing values were imputed by multiple imputation with chained equations using *m* = 100 imputation data sets. *p* values < 0.05 were considered statistically significant.

## Results

Baseline fatigue data were available for 246–270 individuals participating in the tREACH trial. The mean age was 53 years (SD 14.3 years), the DAS score was 4.8, and 67% were females (see Table 1). At baseline, rheumatoid factor and anti-CCP antibodies were present in 73 and 77% of patients, respectively. Erosions were present in 18% of patients (Table 1). The 24 patients that missed their baseline fatigue level worked less often (*p* = 0.05), but did not differ with respect to DAS score (*p* = 0.7), the presence of erosions (*p* = 0.76), or the treatment arm that they had been randomized to (I vs. II *p* = 0.79; I vs. III *p* = 0.90; II vs. III *p* = 0.88) (data not shown).

**Table 1:** Baseline characteristics, total, high fatigued patients, and fatigue and non-fatigued patients

*N* = 246	All patients (*n* = 246)	Fatigued patients (*n* = 113; FAS ≥ 22)	No fatigued patients (*n* = 133; FAS < 22)	*p*
Age, in years^a^	53.3 (14.3)	51.3 (14.1)	55.0 (14.3)	0.04
Sex, female, (%)	68%	75%	62%	0.03^c^
Working status (%)	55%	52%	60%	0.21^c^
Native, Dutch (%)	83%	81%	85%	0.35^c^
Symptom duration (days)	161.5 (88.8)	166.0 (91.0)	158.14 (87.0)	0.48
RF-positive, %	73%	76%	69%	0.01^c^
ACPA-positive, %	77%	76%	80%	0.12^c^
DAS28 (range 0–10)^b^	4.8 (4.0–5.7)	4.9 (4.3–6.0)	4.7 (3.7–5.4)	0.004^d^
Tender joints (range 0–44)^b^	10 (5–15)	11 (6–18)	8 (4–13)	< 0.001^d^
Swollen joints (range 0–44)^b^	8 (4–12)	9 (4–13)	7 (4–11)	0.12^d^
ESR^b^	24 (14–42)	23 (13–44)	24 (15–39)	0.71^d^
VAS global (range 0–100)^b^	53 (34–69)	60 (49–73)	49 (28–63)	< 0.001^d^
VAS fatigue (range 0–100)^b^	53 (31–73)	70 (55–80)	36 (36–54)	< 0.001^d^
FAS (range 10–50)^b^	21 (17–27)	27 (25–31)	17 (15–19)	< 0.001^d^
RADAI (range 0–10)^b^	4.1 (2.8–5.5)	4.7 (3.3–6.0)	3.6 (2.3–4.8)	< 0.001^d^
Coping pain (range 8–32)^b^	15 (11–19)	17 (14–21)	13 (10–16)	< 0.001^d^
Coping limitations (range 8–40)^b^	23 (17–29)	25 (20–30)	21 (16–27)	< 0.001^d^
HADS anxiety (range 0–21)^b^	5 (3–8)	7 (5–10)	4 (2–6)	< 0.001^d^
HADS depression (range 0–21)^b^	4 (2–7)	5 (4–8)	2 (1–4)	< 0.001^d^
Possible case depression [HADS-D ≥ 8, *n*/(%)]	49 (19.9%)	37 (32.7%)	12 (9%)	< 0.001
SF-36 PCS (range 0–100)^b^	39.9 (35.7–44.9)	37.9 (33.4–41.8)	42.0 (37.4–45.9)	< 0.001^d^
SF-36 MCS (range 0–100)^b^	45.4 (41.0–50.5)	43.4 (38.7–48.2)	47.5 (43.7–51.9)	< 0.001^d^
SF-36 vitality (range 0–100)^b^	55.0 (40–70)	40.0 (30–50)	70.0 (55–80)	< 0.001^d^

*RF* rheumatoid factor, *ACPA* anti-citrullinated protein antibodies, *ESR* erythrocyte sedimentation rate, *VAS* Visual Analog Scale, *FAS* Fatigue Assessment Scale, *RADAI* Rheumatoid Arthritis Disease Activity Index, *HADS* Hospital Anxiety and Depression Scale; *SF-36* Short Form 36, *PCS* physical component summary, *MCS* mental component summary

^a^Mean (SD)

^b^Median (IQR)

^c^Pearson’s Chi square

^d^Mann–Whitney *U* test

### Prevalence and pattern of fatigue

At baseline, the mean VAS fatigue score was 51 (SD 26); and the mean FAS score was 22 (SD 7) and 45% of the patients were categorized as fatigued (FAS > 21).

Table 1 summarizes the baseline results for all patients and broken down for the 113 fatigued and 133 non-fatigued patients. Fatigue was most commonly present in younger females. The two fatigue groups differed in disease-related characteristics and patient-reported outcomes (Table 1). Of note, 32% of the fatigued patients reached the cut point of 8 in the HADS that bears clinical relevant levels for depression, compared to 9% of the non-fatigued patients.

**Fig. 1:**
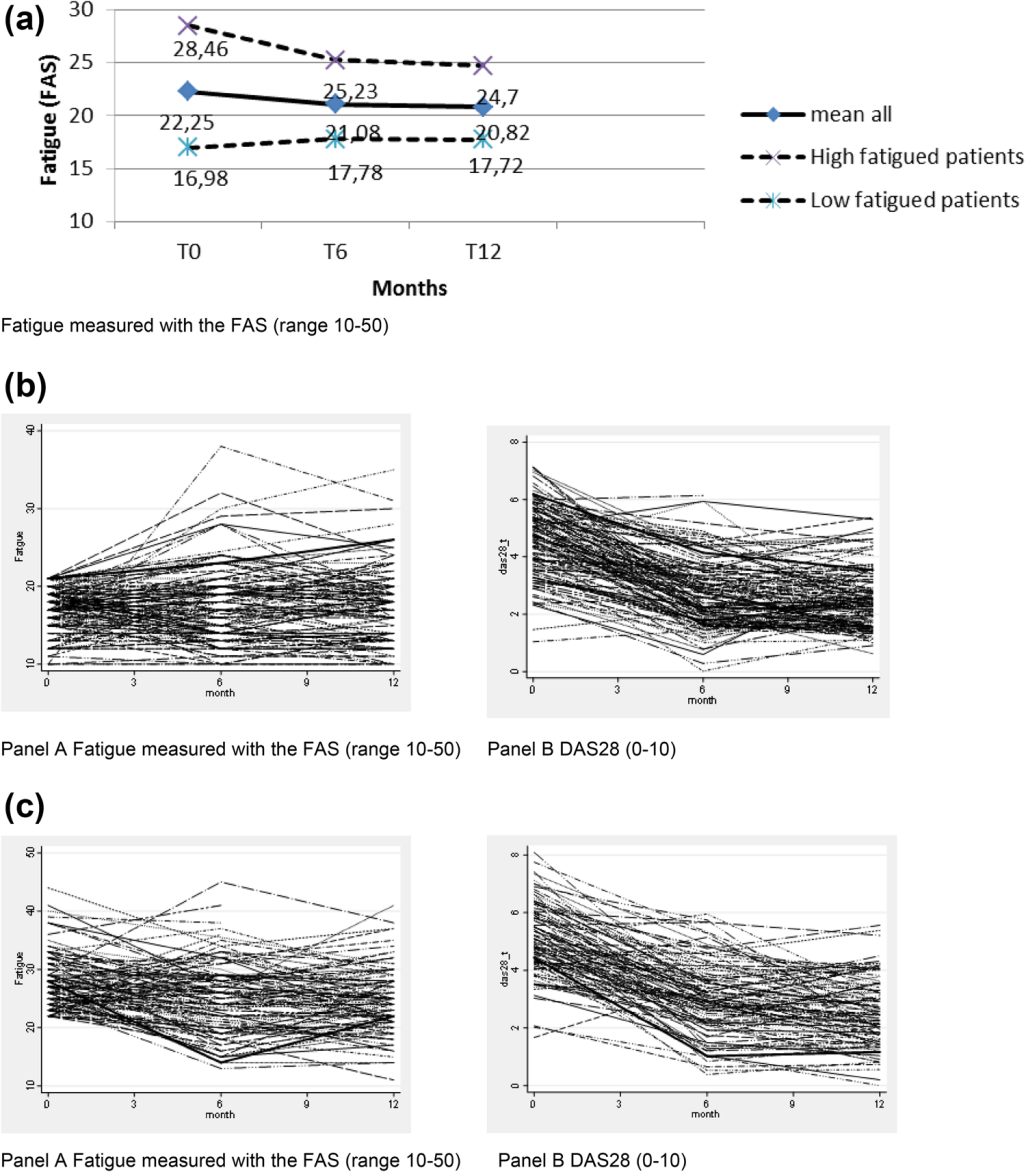
**a** Evolution over time of fatigue. **b** Individual profiles patients with no fatigue—**a** FAS; **b** DAS28. **c** Individual profiles patients with high fatigue—**a** FAS; **b** DAS28

Over time, the FAS fatigue score on average decreased slightly, by 1.4 points in all patients and by 3.8 points in the fatigued patients, while it increased by 0.8 points in the non-fatigued patients (Fig. 1). At 12 months, 43% of all patients were still fatigued. Individual patient profiles showed varying patterns.

Of all fatigued patients (*n* = 113) at baseline, the fatigue level decreased to below the level of no fatigue in only 23%, while 15% of the non-fatigued patients (*n* = 133) became fatigued.

#### Predicting fatigue

**Fig. 2:**
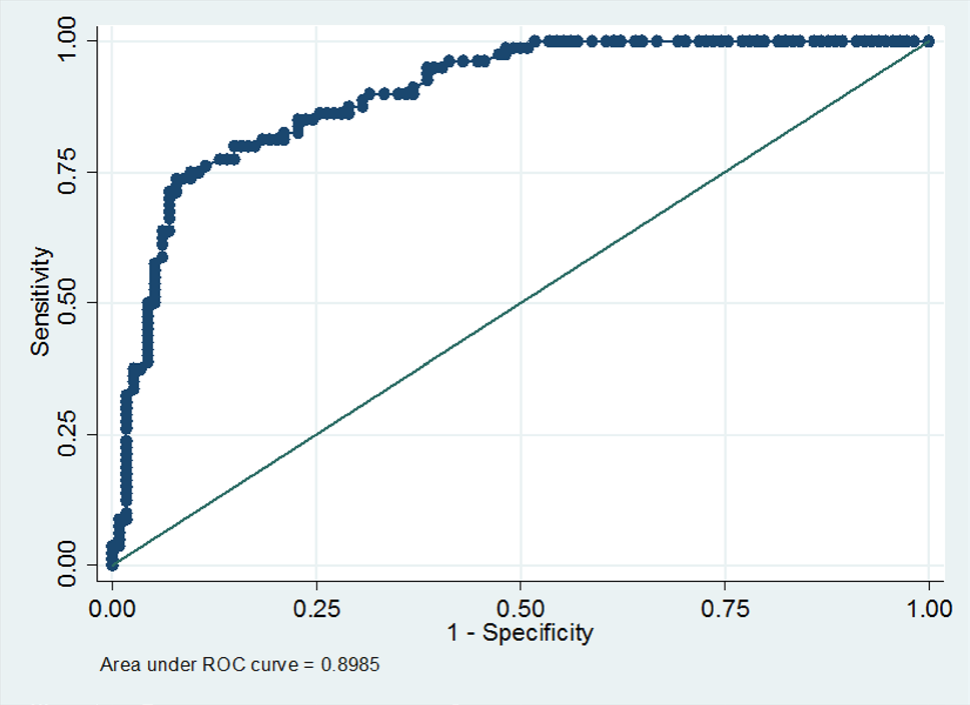
ROC model with only baseline fatigue

The strongest predictor of fatigue was the previous fatigue levels. In an area under curve (AUC) model, baseline fatigue predicted fatigue over time with an AUC of 0.89 (Fig. 2). Adding other variables did not improve the model.

### Factors associated with the strata of fatigue at 12 months

The univariable analysis of patients stratified by baseline fatigue status showed a significantly higher VAS global score in the no fatigue group, as well as a lower score on the Mental Component summary of the SF-36, higher scores on the HADS depression and anxiety, more painful joints, and higher levels of DAS. In the multivariable analysis, only a higher HADS depression score and higher scores on coping with limitations were associated with developing fatigue over time (Table 2).

**Table 2:** Univariable and multivariable analyses for developing fatigue after 12 months for low fatigued and fatigued patients

	Univariable odds ratio (95% CI)		Multivariable odds ratio (95% CI)	
	Fatigue (< 21)	FAS (≥ 22)	Fatigue (< 21)	FAS (≥ 22)
Sex, female	2.60 (0.83 to 8.09)	1.28 (0.48 to 3.40)	3.01 (0.84–10.73)	1.83 (0.65–5.01)
Age, per year	0.98 (0.95 to 1.02)	1.00 (0.97 to 1.04)	0.97 (0.93–1.01)	1.00 (0.97–1.04)
Education	1.01 (− 0.03 to 2.07)	− 0.03 (− 1.08 to 1.01)		
Working status (Y/N)	1.89 (0.67 to 5.30)	1.28 (0.53 to 3.06)		
Nationality Natively/Dutch	1.33 (0.29 to 6.01)	2.98 (0.89 to 9.95)	7.45 (0.74–74.83)	3.43 (0.99–11.82)*
DAS28	1.96 (1.07 to 3.59) *	1.45 (0.93 to 2.25)		
ESR	1.007 (0.98 to 1.02)	1.00 (0.98 to 1.02)		
Tender joints (0–44)	1.12 (1.01 to 1.23)**	1.03 (0.96 to 1.10)		
Swollen joints (0–44)	1.03 (0.95 to 1.12)	1.02 (0.96 to 1.08)		
VAS global (0–100)	1.02 (1.00 to 1.05)**	1.01 (0.99 to 1.04)		
Radai (0–10)	1.19 (0.88 to 1.61)	0.97 (0.75 to 1.25)		
Hads depression (0–21)	1.20 (1.03 to 1.40)**	1.04 (0.91 to 1.19)	1.33 (1.08–1.62)**	
Hads anxiety (0–21)	1.15 (1.01 to 1.32)*	0.98 (0.86 to 1.12)		
Coping limitations (8–40)	1.06 (0.99 to 1.14)	0.98 (0.92 to 1.04)	1.09 (1.00–1.18)*	
Coping pain (8–32)	1.08 (0.98 to 1.19)	0.96 (0.87 to 1.05)		
Physical health (SF-36, 0–100)	0.94 (0.86 to 1.02)	1.00 (0.94 to 1.07)		
Mental health (SF-36, 0–100)	0.87 (0.80 to 0.96)**	0.98 (0.92 to 1.05)		

Multivariable analysis corrected for sex and age. Level of significance *p = 0.05/**p = 0.01/***p = 0.001 cut point for FAS ≤ 21 non-fatigue > 22 fatigued

*ESR* erythrocyte sedimentation rate, *VAS* Visual Analog Scale, *RADAI* Rheumatoid Arthritis Disease Activity Index, *HADS* Hospital Anxiety and Depression Scale, *SF-36* Short Form 36, *PCS* physical component summary, *MCS* mental component summary

In the fatigued patients, no factors apart from the severity of fatigue itself explained the recovery of fatigue in the univariable and multivariable analyses (Table 2).

## Discussion

In this study, almost half of the early RA patients were fatigued over the first year after diagnosis, although they had been treated by an early, intensive, and tight-controlled strategy. Of those who had no fatigue at baseline, 15% became fatigued, while most of those who were fatigued at baseline (77%) remained fatigued despite lesser disease activity. The minor change in fatigue levels was also reflected in the AUC analysis of all patients, which showed that baseline level of fatigue was the strongest factor in predicting follow-up levels of fatigue. This factor was so strong that adding other variables did not improve the model. In a stratified analysis among the non-fatigued patients at baseline, higher levels on the HADS were associated with higher levels of fatigue later on.

The literature on the course of fatigue in early RA has been conflicting. A cohort study in early RA patients reported recovery of fatigue over time [14]. Recovery over time is more often observed in studies evaluating biological treatment with longstanding RA patients [34, 35]. However, another cohort study showed persistent fatigue over time with almost no change since diagnosis [13]. Moreover, a recent meta-analysis found that treatment with biologicals only led to a small, but statistically significant, improvement in levels of fatigue [36]. Since our study was performed in an early RA population treated with induction by conventional DMARDs, it is interesting to see a similar pattern, with fatigue decreasing by only 6% over 1 year of follow-up.

At baseline, both inflammatory disease characteristics and patient-reported characteristics were more pronounced in the fatigued than in the non-fatigued patients. It is not clear; therefore, whether fatigue in the present study was related to the disease or to other more personal characteristics. Some direct and indirect observations suggest a less prominent relationship with the disease itself. Over time, disease activity decreased, while fatigue remained present in most patients. In the initially non-fatigued patients, the presence of symptoms related to depression and anxiety, more painful joints, lower scores on the Mental Component summary of the SF-36, and higher levels of DAS were predictive in the univariate analysis for the development of fatigue. This may suggest that other pre-existing factors contribute to the presence of fatigue, of which depression/anxiety is the most powerful relation. Moreover, the relation of depression in the fatigued patients was also pronounced at baseline. Depression seems to interplay with fatigue in our early RA study population. Depressive symptoms are a common feature of both established and early RA [37] and are associated with fatigue [38]. The direction of the association, thus is depression induced by fatigue or fatigue induced by depression, is under debate. According to Druce et al., both directions are possible [39]. A dynamic conceptual model of RA fatigue showed the bi-directional relation for depression and fatigue [40]. Irrespective of the direction of this relation, the high levels of symptoms related to depression warrant monitoring over time and further examination by a psychologist if symptoms persist.

Several aspects of this study need further discussion. There are many ways to analyse fatigue over time. We used simple logistic regression and the AUC, but also considered longitudinal models taking into account individual patient profiles. These models did not lead to different results and insights than described in the analysis presented here.

In 2003, it had been decided to use the FAS and the VAS fatigue, at a time when not many specific RA fatigue instruments were available. The FAS has a good internal consistency reliability and validity [24, 25]. The lack of a standardized VAS cut-off point for high and low fatigue prevented a clear interpretation of the VAS fatigue scores. We were able to analyse a substantial number of covariates influencing fatigue, but data on, for example, sleep quality or the presence of symptoms of fibromyalgia were not available. Strong points of this study include its longitudinal design and the use of data of the protocolled medication and tight-controlled treatment. As this study was not an RCT, we could study the longitudinal evolvement of fatigue and the development of fatigue among those patients with initial low fatigue level at baseline and recovery of fatigue among those with initial high level of fatigue. Medication had no effect on the decrease of fatigue in both groups (data not shown).

Given that many patients in this study in early RA showed fatigue, it seems advisable to quantify fatigue and depression in daily care. Our results and those of others suggest that fatigue does not resolve by itself. To facilitate (more) self-management behaviour, it is important to inform patients about the course of fatigue. Nurses are ideally suited to address this topic during consultations.

Screening on fatigue and depressive symptoms at baseline and follow-up will make a patient feel that his or her fatigue is acknowledged, and may also improve patient satisfaction and treatment outcomes.

## Conclusion

Despite a strict treat-to-target strategy in early RA patients, fatigue is and remains a problem for many patients. Initial fatigue level is the main predictor for fatigue at follow-up. Higher levels of depression were associated with developing fatigue in initially non-fatigued patients. Discussing and monitoring fatigue and depression at baseline and follow-up might be important to acknowledge its presence and improve patients’ self-management of fatigue. Referral to a psychologist may be indicated.
